# Pyrrolidinyl Synthetic Cathinones α-PHP and 4F-α-PVP Metabolite Profiling Using Human Hepatocyte Incubations

**DOI:** 10.3390/ijms22010230

**Published:** 2020-12-28

**Authors:** Jeremy Carlier, Xingxing Diao, Raffaele Giorgetti, Francesco P. Busardò, Marilyn A. Huestis

**Affiliations:** 1 Chemistry & Drug Metabolism Section, Intramural Research Program, National Institute on Drug Abuse, National Institutes of Health, Baltimore, MD 21224, USA; jerem.carlier@gmail.com (J.C.); xxdiao@simm.ac.cn (X.D.); marilyn.huestis@gmail.com (M.A.H.); 2 Department of Excellence of Biomedical Sciences and Public Health, Unit of Forensic Toxicology, Section of Legal Medicine, Marche Polytechnic University, 60126 Ancona, Italy; r.giorgetti@staff.univpm.it; 3 Department of Anatomical, Histological, Unit of Forensic Toxicology, Section of Legal Medicine, Forensic, and Orthopedic Sciences, Sapienza University of Rome, 00198 Rome, Italy; 4 Shanghai Institute of Materia Medica, Chinese Academy of Sciences, Shanghai 201203, China; 5 Institute of Emerging Health Professions, Thomas Jefferson University, Philadelphia, PA 19107, USA

**Keywords:** α-PHP, PV7, 4F-α-PVP, novel psychoactive substance, synthetic cathinone, bath salt, hepatocyte metabolism, liquid chromatography–high resolution tandem mass spectrometry

## Abstract

For more than ten years, new synthetic cathinones (SCs) mimicking the effects of controlled cocaine-like stimulants have flooded the illegal drug market, causing numerous intoxications and fatalities. There are often no data on the pharmacokinetics of these substances when they first emerge onto the market. However, the detection of SC metabolites is often critical in order to prove consumption in clinical and forensic settings. In this research, the metabolite profile of two pyrrolidinyl SCs, α-pyrrolidinohexaphenone (α-PHP) and 4′′-fluoro-α-pyrrolidinovalerophenone (4F-α-PVP), were characterized to identify optimal intake markers. Experiments were conducted using pooled human hepatocyte incubations followed by liquid chromatography–high-resolution tandem mass spectrometry and data-mining software. We suggest α-PHP dihydroxy-pyrrolidinyl, α-PHP hexanol, α-PHP 2′-keto-pyrrolidinyl-hexanol, and α-PHP 2′-keto-pyrrolidinyl as markers of α-PHP use, and 4F-α-PVP dihydroxy-pyrrolidinyl, 4F-α-PVP hexanol, 4F-α-PVP 2′-keto-pyrrolidinyl-hexanol, and 4F-α-PVP 2′-keto-pyrrolidinyl as markers of 4F-α-PVP use. These results represent the first data available on 4F-α-PVP metabolism. The metabolic fate of α-PHP was previously studied using human liver microsomes and urine samples from α-PHP users. We identified an additional major metabolite (α-PHP dihydroxy-pyrrolidinyl) that might be crucial for documenting exposure to α-PHP. Further experiments with suitable analytical standards, which are yet to be synthesized, and authentic specimens should be conducted to confirm these results.

## 1. Introduction

Synthetic cathinones (SCs), or bath salts, are novel psychoactive substances (NPSs) designed to induce cocaine-like stimulant effects while evading legislation and analytical detection. SCs inhibit the transport of monoamines in the central nervous system, with specific affinities for dopamine, norepinephrine, and serotonin transporters (DAT, NET, and SERT, respectively), inducing euphoria and increased energy, but also tachycardia, elevated blood pressure, hyperthermia, agitation, delirium, psychosis, and death. SC use accounts for many intoxications and deaths, mainly through cardiac arrest or multi-organ failure [1,2]. SC effects are closely related to their specific selectivity for DAT, NET, and SERT. Particularly, DAT/SERT inhibition is associated with distinct psychoactive effects and abuse liability. For example, methedrone and 4-ethylmethcathinone have DAT/SERT inhibition ratios similar to that of 3′′,4′′-methylenedioxymethamphetamine (MDMA), whereas methylenedioxypyrovalerone (MDPV) and pyrovalerone have a much higher ratio, resulting in higher reinforcing effects and abuse potential [3].

**Figure 1:**
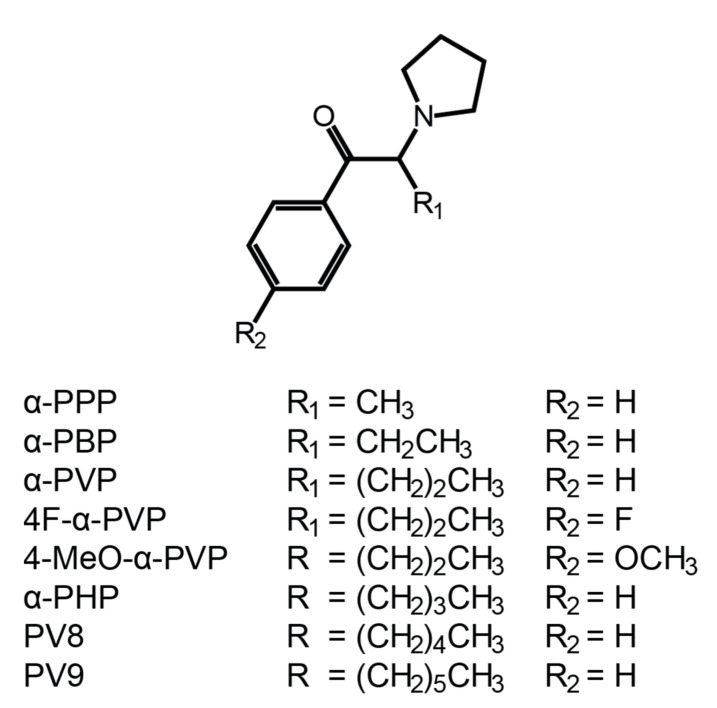
Chemical structures of α-PHP, 4F-α-PVP, and structural analogues α-PPP, α-PBP, α-PVP, 4-MeO-α-PVP, PV8, and PV9.

α-PHP (or PV7 or α-pyrrolidinohexaphenone, 1-phenyl-2-(1′-pyrrolidinyl)-1-hexanone) and 4F-α-PVP (or 4′′-fluoro-α-pyrrolidinovalerophenone, 1-(4′′-fluorophenyl)-2-(1′-pyrrolidinyl)-1-pentanone) are pyrrolidinyl SCs, characterized by the presence of a butyl chain and a phenyl for α-PHP, and a propyl chain and a *para*-fluoro-phenyl ring for 4F-α-PVP (Figure 1). Both SCs were first identified in 2014 in material seized in the Japanese illegal drug market or for sale over the Internet [4,5]. α-PHP was recently involved in several combined drug intoxication fatalities [6,7,8,9,10]. All the cases involved the co-ingestion of other SCs, mainly α-pyrrolidinovalerophenone (α-PVP) and 3′′,4′′-methylenedioxy-α-pyrrolidinohexiophenone (MDPHP), or often opiates and/or benzodiazepines. A total of 13 α-PHP-related deaths were reported to the early warning system of the European Monitoring Centre for Drugs and Drug Addiction (EMCDDA) between 2017 and 2020 [2], with additional cases reported to the United Nations Office on Drugs and Crime (UNODC) early warning advisory system [11]. No intoxication cases or deaths involving 4F-α-PVP have been reported to date. Halogenated SCs inhibit SERT with a higher potency than that of their non-halogenated analogues, inducing potentially fatal serotonin syndrome effects—tachycardia; nausea; hyperthermia; rhabdomyolysis; psychomotor tremors; and liver, kidney, and lung failure [12,13,14,15]. Therefore, 4F-α-PVP is expected to demonstrate higher toxicity than α-PVP, which has been involved in many intoxications and fatalities [1,2]. Halogenated SC use recently became popular, likely due to its higher potency [2], and 4F-α-PVP use is also expected to increase. In December 2019, the World Health Organization (WHO) recommended the control of α-PHP under Schedule II of the UNODC convention on psychotropic substances of 1971 [16]. It is currently controlled under Class B in the United Kingdom; Schedule I in the United States; is illegal in China, Sweden, Poland, and Italy; and is classified as a narcotic in Japan [11,17]. 4F-α-PVP is illegal in China and classified as a designated chemical substance in Japan [17].

Exposure to α-PHP can be determined through the detection of parent drugs in biological specimens [6,7,8,9]. α-PHP has been quantified in post-mortem cases with concentrations ranging from 4 to 52 ng/mL in blood [6,7], 5.6 ng/mL in urine [9], 3.5 to 83.3 ng/g in tissues [9], and 4700 pg/mg in hair [8]. Although α-PHP was not the primary cause of death in these cases, active and toxic concentrations are expected to be low. 4F-α-PVP concentrations in biological specimens have not been reported to date. In clinical and forensic settings, toxicologists often focus on the detection of drug metabolites in urine, which are often detected in higher concentrations and over an extended time course. A prerequisite for the detection of metabolites is the characterization of the drug’s metabolic fate. However, there are often no data on SC pharmacokinetics when they first emerge onto the illegal drug market. To date, there are no data available on 4F-α-PVP pharmacokinetics. α-PHP metabolism, however, was investigated using human liver microsomes and authentic urine samples. Several α-PHP metabolites were identified, mainly involving ketoreduction, pyrrolidinyl oxidation, and N-dealkylation to *N*-butanoic acid or *N*-hydroxy-4′-ketobutyl [18]. Reduction of the ketone group is a prominent transformation in SC metabolism [19,20,21,22,23], but it is not detected in human liver microsomes and human S9 fractions [24,25,26]. In vitro incubations with human hepatocytes have proved suitable for the prediction of NPS metabolic fate in previous studies [27,28,29,30,31,32,33,34]. Additionally, it has proven particularly accurate for the prediction of urinary metabolites of structural analogues α-pyrrolidinoheptaphenone (PV8) and α-pyrrolidinopentiothiophenone (α-PVT) (Figure 1), suggesting an important hepatic metabolism of pyrrolidinyl SCs [21,22]. Combining human hepatocyte incubations and the analysis of urine samples from authentic users seems therefore the best strategy to identify optimal SC use markers. Considering the potential health threat of α-PHP and 4F-α-PVP, we investigated their metabolite fate using pooled human hepatocyte incubations, liquid chromatography–high-resolution tandem mass spectrometry (LC-HRMS/MS), and data-mining software, with the aim of identifying suitable markers of consumption.

## 2. Results and Discussion

### 2.1. α-PHP and 4F-α-PVP MS/MS Fragmentation Patterns

**Figure 2:**
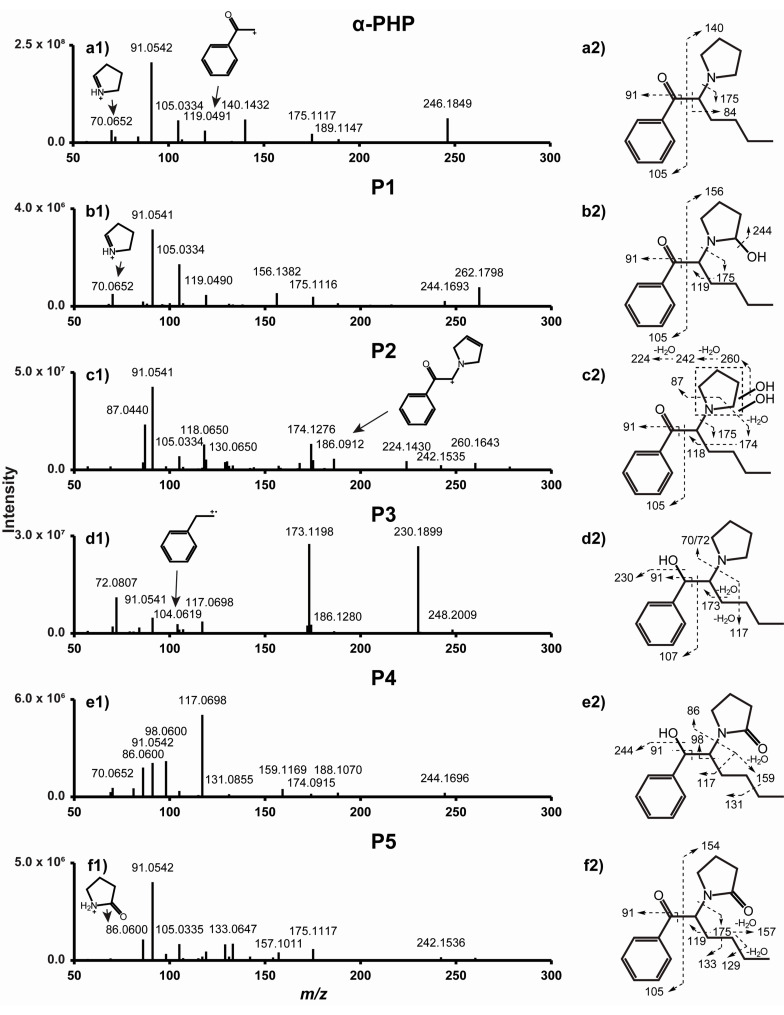
Product ion spectra of α-PHP (**a1**) and metabolites P1 (**b1**), P2 (**c1**), P3 (**d1**), P4 (**e1**), and P5 (**f1**), and their postulated fragmentation patterns (**a2**–**f2**, respectively). Dashed arrow indicates fragmentation site.

**Figure 3:**
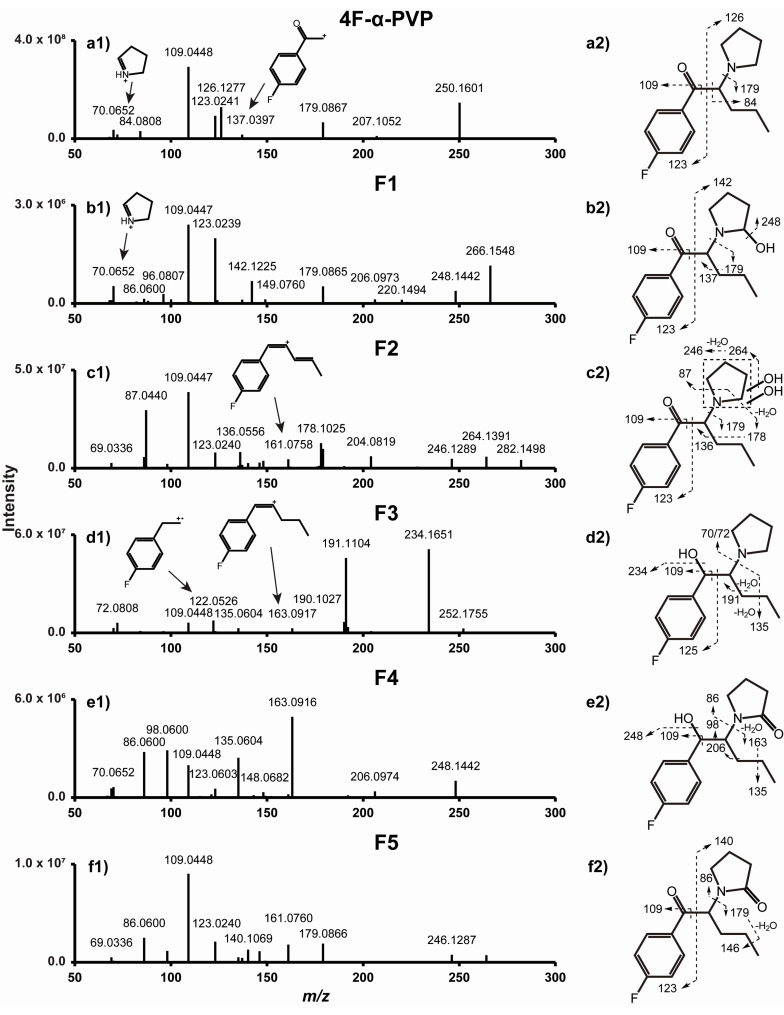
Product ion spectra of 4F-α-PVP (**a1**) and metabolites F1 (**b1**), F2 (**c1**), F3 (**d1**), F4 (**e1**), and F5 (**f1**), and their postulated fragmentation patterns (**a2**–**f2**, respectively). Dashed arrow indicates fragmentation site.

As expected from two structural analogues, α-PHP and 4F-α-PVP display similar fragmentation patterns (Figure 2 and Figure 3). Major fragments are produced through C-C cleavage at the α carbons of the carbonyl and pyrrolidinyl groups, yielding fragments *m*/*z* 105.0334 and 140.1432 from α-PHP, and *m*/*z* 123.0241 and 126.1277 from 4F-α-PVP. Further fragmentation and rearrangement yield the characteristic tropylium ion from α-PHP (*m*/*z* 91.0542), the fluorotropylium ion from 4F-α-PVP (*m*/*z* 109.0448), and the pyridinium ion from both molecules (*m*/*z* 84.0808). C-C cleavage at the pyrrolidinyl α carbon also produces fragments *m*/*z* 175.1117 and 189.1147 for α-PHP, *m*/*z* 179.0867 and 207.1052 for 4F-α-PVP, and *m*/*z* 72.0808 for both. Further fragmentation produces fragments *m*/*z* 119.0491 and 137.0397 from fragments *m*/*z* 175.1117 and 179.0867, respectively. McLafferty rearrangement through the ionization of the oxygen atom may occur, yielding fragment *m*/*z* 70.0652 from α-PHP and 4F-α-PVP. α-PHP fragmentation matches well with that reported by Matsuta et al., although the instrument employed was different [35]. 4F-α-PVP MS/MS fragmentation was described by Uchiyama et al. by gas chromatography (GC)-MS, but the authors only identified two fragments, i.e., *m*/*z* 95 (attributed to the fluorophenyl group) and 126 [5].

### 2.2. α-PHP Metabolism with Human Hepatocytes

**Figure 4:**
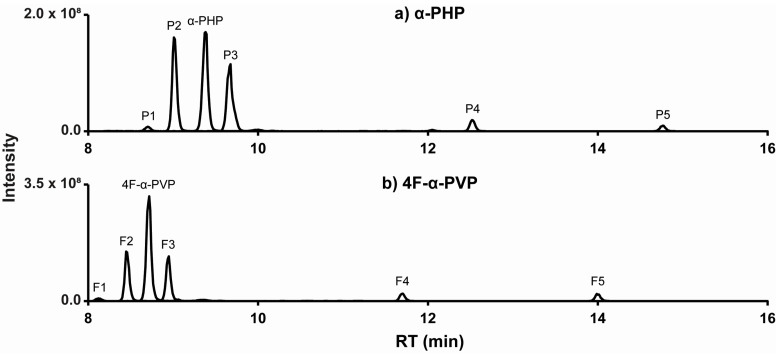
Combined extracted ion chromatogram of α-PHP (**a**), 4F-α-PVP (**b**), and metabolites obtained from 3 h hepatocyte incubation. Mass tolerance, 5 ppm; RT, retention time.

The α-PHP signal decreased to 21% after 3 h incubation with hepatocytes, generating a total of 24 metabolites. Five metabolites totaling more than 95% of the total metabolite peak area are described in this article are listed from P1 to P5 by ascending retention time (Figure 4). All metabolites were absent from controls, ruling out interferences and non-enzymatic reactions.

**Figure 5:**
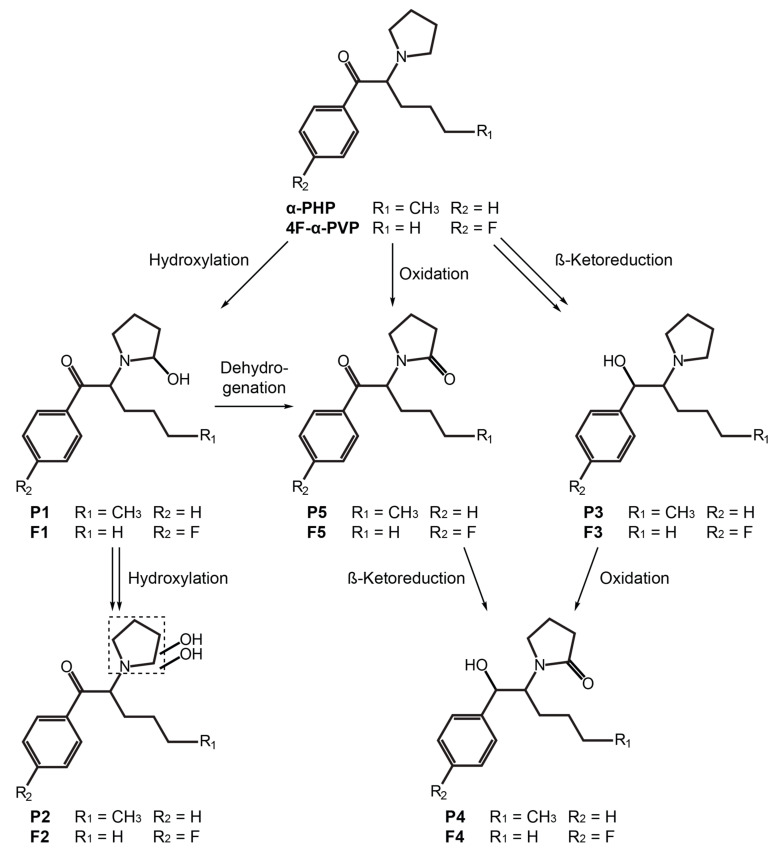
Postulated metabolic pathway of α-PHP and 4F-α-PVP. Double arrow indicates major pathway.

Major metabolic reactions included reduction of the ketone group (P3 and P4) and hydroxylation (P1 and P2) and oxidation (P4 and P5) of the pyrrolidinyl ring. Dealkylation to the primary amine and alkyl hydroxylation were identified as minor phase I metabolites. α-PHP alkyl chain transformations were less frequent than those of structural analogues with a longer chain [20,21]. However, Manier et al. reported that α-PHP was mainly hydroxylated at the pyrrolidinyl ring [26], in accordance with our results. In fact, the length of the alkyl chain seems to strongly influence the metabolic pathway of analogues [35]. Phase II glucuronidations were also rare, as observed in the metabolic profile of analogues (in vitro and in vivo) [21,22] and that of α-PHP in the urine of several users [18]. Therefore, hydrolysis may be not a prerequisite for the detection of α-PHP and metabolites in urine. The accurate mass molecular ion, retention time, elemental composition, nominal mass for diagnostic product ions, and mass spectrometry peak areas (extracted ion chromatogram) for P1–P5 are reported in Table 1. The fragmentation pattern of P1–P5 is displayed in Figure 2. α-PHP’s metabolic pathway is proposed in Figure 5.

#### 2.2.1. β-Ketoreduction

Reduction of the α-PHP ketone group (+2H) resulted in the formation of P3 (α-PHP hexanol), as suggested by a +2.0157 Da mass shift from parent and a water loss (*m*/*z* 230.1899) detected in P3’s product-ion spectrum. The signal intensity of the water loss was particularly intense, as it is favored by the proximity of the phenyl group. The low intensity of ion *m*/*z* 70.0652 compared to *m*/*z* 72.0807), and the absence of ion *m*/*z* 177.1274 (*m*/*z* 175.1117 in α-PHP) suggested that McLafferty rearrangement did not occur, due to the reduction of the ketone group. Instead, ion *m*/*z* 173.1198, produced by C-C cleavage at the α carbon of the pyrrolidinyl group and a water loss, was intense. The presence of the tropylium ion further indicated that the phenyl group did not carry the transformation. β-ketoreduction is a prominent transformation in SC metabolism [19,20,21,22,23], and P3 was indeed one of the metabolites with the most intense signal in our experiments. It was also detected as a major metabolite by Paul et al. and Matsuta et al. in urine samples from α-PHP users [18,35].

Reduction of the ketone group implies the formation of a chiral center and two diastereoisomers, depending on hydroxyl positioning. Matsuta et al. determined that the formation of one isomer is favored over the other (ten-times higher concentration), but they could not distinguish the two molecules [35]. Most probably, the two metabolites coeluted in our experiments, which is not problematic considering that HRMS/MS is not suitable to identify the two isomers.

#### 2.2.2. Pyrrolidinyl Hydroxylation

P1 (α-PHP 2′-hydroxy-pyrrolidinyl) was formed by hydroxylation (+O), as suggested by a +15.9950 Da mass shift from the parents and a water loss (*m*/*z* 244.1693) detected after fragmentation. The presence of ions *m*/*z* 91.0542, 105.0335, and 175.1117, also detected in the α-PHP product-ion spectrum, indicated that the transformation occurred at the pyrrolidinyl ring. Ion *m*/*z* 156.1382 (*m*/*z* 140.1432 in α-PHP) also suggested that the hydroxylation occurred either at the pyrrolidinyl ring or the alkyl chain of the molecule. The exact position of the hydroxylation could not be determined. However, the metabolic profile of structural analogues α-PVP and α-PBP strongly suggests that the hydroxylation may have occurred at position 2′ of the pyrrolidinyl ring, forming a hemiaminal group that can be an intermediate to P4 and P5 (Section 2.2.3.) [36,37,38].

P2 (α-PHP dihydroxy-pyrrolidinyl) was the metabolite with the most intense signal detected in our experiments. The +31.9898 Da mass shift from α-PHP and the two water losses (*m*/*z* 260.1643 and 242.1535) suggested a dihydroxylation (+2O). Like P1, ions *m*/*z* 91.0541, 105.0334, and 175.1117 indicated that the transformations occurred at the pyrrolidinyl ring. Pyrrolidinyl dihydroxylation was also a major transformation in the metabolic pathway of 4-methoxy-α-PVP and α-PVT, two structural analogues, using human hepatocytes and urine samples from users [22,23]. P2’s molecular mass and theoretical formula also match those of α-PHP *N*-butanoic acid, which could theoretically be formed by γ-lactam formation, then N-dealkylation and further carboxylation of the pyrrolidinyl ring, as reported in the metabolism of SCs with an analogous structure [26]. However, the absence of carboxylic acid loss and the presence of two water losses in the P2 fragmentation pattern rather indicate a dihydroxylation. Additionally, Vickers and Polsky determined that further transformation following lactam formation (Section 2.2.3.) is unlikely in the metabolism of N-heterocycles [36]. Lactam opening was observed in the metabolism of nicotine, an extensively studied molecule with a pyrrolidinyl substructure, but it was only a minor metabolic pathway [39]. Paul et al. identified two major α-PHP metabolites with P2 molecular mass in the urine of an authentic user, which they attributed to the formation of *N*-butanoic acid and *N*-hydroxy-4′-oxobutane (M13 or M14, respectively, in the article), both formed by lactam formation and opening of the N-heterocycle [18]. They correctly located the position of the transformations on the pyrrolidinyl ring of the molecule (fragments *m*/*z* 91.0542 and 175.1117). Like P2 in our experiments, the authors did not detect a carboxylic acid loss in the M13 and M14 fragmentation patterns, but they detected one (M13) or two (M14) water losses instead. They also detected further minor metabolites with subsequent hydroxylation and dealkylation. We believe that these data rather point towards two different dihydroxylations at the pyrrolidinyl ring, as identified in the present study (P2). Comparison with suitable analytical standards, which are yet to be synthesized, would bring a definite answer. In another article, Matsuta et al. did not identify P1 and P2 during their investigation of α-PHP metabolism using the urine of five authentic users, as they mainly focused on the metabolic transformations at the alkyl chain [35]. The characterization of P2, a potential major metabolite that was not reported in previous studies, might be crucial for documenting α-PHP use.

Manier et al. determined the kinetics of α-PHP hydroxylations using human liver microsomes and S9 human fractions [26]. They determined that cytochrome P450 (CYP) 2C9 is mainly involved in the hydroxylation of α-PHP (71% of reactions with selected enzymes); CYP2C19, CYP3A4, and CYP2B6 catalyzed 18%, 6%, and 5% of reactions, respectively.

#### 2.2.3. Oxidation

Pyrrolidinyl oxidation or hydroxylation and dehydrogenation (+O –2H) occurred in P5 (α-PHP 2′-keto-pyrrolidinyl), as indicated by a +13.9794 Da mass shift from the parents and the detection of α-PHP fragments *m*/*z* 91.0542, 105.0335, and 175.1117. P5’s late elution compared to parents supported an oxidation at position 2′ of the pyrrolidinyl ring (γ-lactam), which acts as a hindrance for hydrogen bonding [22]. In addition, 2′-pyrrolidinyl oxidation is another transformation that is predominant in the metabolism pyrrolidinyl-containing molecules through CYP metabolism [36] and pyrrolidinyl SCs [19,20,21,22,23,24,26], and it was also detected as a major metabolite by Paul et al. and Matsuta et al. in urine samples from α-PHP users [18,35]. Fragment *m*/*z* 86.0600 may also be explained by another McLafferty rearrangement through the ionization of the newly formed amide group.

Following the same reasoning, we found that P4 (α-PHP 2′-keto-pyrrolidinyl-hexanol) was formed by oxidation at the pyrrolidinyl ring (+O –2H), as observed in P5, and a β-ketoreduction (+2H), as observed in P3 (Section 2.2.1.).

### 2.3. 4F-α-PVP Metabolism with Human Hepatocytes

The 4F-α-PVP signal decreased to 29% after 3 h incubation with human hepatocytes. A total of 11 metabolites were identified, with five molecules totaling more than 97% of the total metabolite peak area, which were listed from F1 to F5 by ascending retention time (Figure 4). All metabolites were absent in controls.

4F-α-PVP’s major transformations were the same as those of α-PHP, with similar relative intensities (Table 1). Fewer minor metabolic reactions were detected, and no transformations of the alkyl chain were identified, in consistency with the results of Matsuta et al. on the influence of the alkyl chain length on the metabolic profile of analogues [35]. Several alkyl chain transformations, however, were observed in α-PVP metabolism using human hepatic microsomes and controlled administrations to rats [24]. No phase II transformations were detected either. The fragmentation pattern of F1–F5 is displayed in Figure 3. 4F-α-PVP’s metabolic pathway is proposed in Figure 5.

#### 2.3.1. β-Ketoreduction

Unsurprisingly, β-ketoreduction (+2H) appeared as a major metabolic transformation in our experiments, leading to F3 formation (4F-α-PVP hexanol). F3 fragmentation and fragment relative intensities were very similar to those of P3 (Section 2.2.1.), with fragment mass shifts depending on the fluorine atom and the alkyl side chain. Like α-PHP, mass shift from parent (+2.0157 Da), and ions *m*/*z* 72.0808, 109.0448, 191.1104, and 234.1651 allowed for F3 identification. β-ketoreduction is also a major metabolic transformation of α-PHP (Section 2.2.1.) and other SCs such as α-PBP, α-PVP, and α-PVT [19,20,21,22,23].

#### 2.3.2. Pyrrolidinyl Hydroxylation

Pyrrolidinyl hydroxylation (+O) also occurred in 4F-α-PVP, producing F1 (4F-α-PVP 2′-hydroxy-pyrrolidinyl). Like F3, F1’s fragmentation pattern and fragment relative intensities were similar to those of P1 (Section 2.2.2.). The mass shift from parents (+15.9949 Da) and ions *m*/*z* 109.0447, 123.0239, 142.1225, 179.0865, and 248.1442 indicated a hydroxylation at the pyrrolidinyl ring. As suggested by the metabolic profile of structural analogues α-PVP and α-PBP, the reaction probably occurred at position 2′ of the pyrrolidinyl ring, forming a hemiaminal group that can be an intermediate for F4 and F5 formation (Section 2.3.3.) [37,38].

Like α-PHP and other SCs [22,23], pyrrolidinyl dihydroxylation (+2O) was a major 4F-α-PVP metabolic transformation. F2 (4F-α-PVP dihydroxy-pyrrolidinyl) was identified through the +31.9898 Da mass shift from the parents and ions *m*/*z* 109.0447, 123.0240, and 179.0867, similar to α-PHP (Section 2.2.2.). Although the opening and carboxylation of the pyrrolidinyl ring was observed in the metabolism of several SCs with a similar structure [26], the absence of carboxylic acid and the presence of two water losses (*m*/*z* 264.1391 and 246.1289) in the F2 fragmentation pattern indicated a dihydroxylation.

#### 2.3.3. Oxidation

Another frequent metabolic transformation of pyrrolidinyl SCs is the formation of a γ-lactam [19,20,21,22,23,24,26]. The F5 (4F-α-PVP 2′-keto-pyrrolidinyl) retention time shift and the +13.9793 Da mass shift from 4F-α-PVP, and ions *m*/*z* 109.0448, 123.0240, and 179.0866 indicated a formation of a ketone at position 2′ of the pyrrolidinyl ring, similar to α-PHP (Section 2.2.3.).

F4 (4F-α-PVP 2′keto-pyrrolidinyl-hexanol) was a combination of β-ketoreduction (+2H) and pyrrolidinyl oxidation (+O –2H).

### 2.4. Optimal Targets for α-PHP and 4F-α-PVP

The four α-PHP metabolites with the most intense signal intensity detected after 3 h incubation with human hepatocytes were α-PHP dihydroxy-pyrrolidinyl (P2), α-PHP hexanol (P3), α-PHP 2′-keto-pyrrolidinyl-hexanol (P4), and α-PHP 2′-keto-pyrrolidinyl (P5), with P2 and P3 representing almost 85% of the total metabolite peak area. Similarly, the four 4F-α-PVP metabolites with the most intense signal intensity were 4F-α-PVP dihydroxy-pyrrolidinyl (F2), 4F-α-PVP hexanol (F3), 4F-α-PVP 2′-keto-pyrrolidinyl-hexanol (F4), and 4F-α-PVP 2′-keto-pyrrolidinyl (F5), with F2 and F3 representing more than 80% of the total metabolite peak area. To the best of the authors’ knowledge, these metabolites were not observed in the metabolism of other SCs and can be used as targets for α-PHP and 4F-α-PVP use. α-PiHP is an α-PHP isomer with a 4-methyl-pentanone structure instead of a hexanone, which was recently identified in seized materials [40]. α-PiHP possesses the same molecular formula and mass as those of α-PHP and the identification of the two SCs could be problematic. Although the metabolic fate of α-PiHP has not yet been studied, the alkyl chain of α-PHP major metabolites remained untransformed, and it is unlikely to observe P2, P3, P4, or P5 in α-PiHP metabolism. Similarly, α-PHP major metabolites are unlikely to be found in the metabolism of pyrovalerone, another isomer [41].

Glucuronide and sulfate hydrolysis are not required for PHP and 4F-α-PVP urinalysis due to the low incidence of phase II transformations.

### 2.5. Analytical Considerations

GC separation should be avoided for SC testing and metabolite identification, considering their thermal degradation through oxidative decomposition [42]. This decomposition was also observed with α-PVP, most likely resulting in formation of an enamine [43]. Although derivatization can increase SC thermal stability, pyrrolidinyl SCs are not derivatized with common reagents, and the derivatization of metabolites is not guaranteed [42]. Additionally, small molecules and metabolites may be unstable at high temperatures during derivatization or GC testing [44]. LC-MS/MS is typically used for metabolite identification studies, and HRMS is popular for identifying expected and unexpected metabolites with accurate-mass capabilities. We employed a long chromatographic run to best separate isomers and limit matrix effects. We selected a polar end-capped C_18_ column and a mobile phase gradient, as previously reported in other SC metabolite identification studies [21,22,23]. The lack of a buffer limited interference and ammonium, sodium, and potassium adducts. Metabolites are generally more polar than parent drugs to favor elimination. However, SC metabolites with γ-lactam formation often appeared to elute later than parents in reversed-phase chromatography [18,21,22,23,35]. Therefore, we ensured that αPHP and 4F-α-PVP approximately eluted mid-gradient to best separate the metabolites.

MS source parameters were optimized using α-PHP and 4F-α-PVP reference standards in an attempt to achieve good signals. However, the behavior of metabolites in these conditions is hardly predictable. Similarly, a ramped collision energy generated many specific fragments to allow metabolite identification, but optimal collision energy may differ from one metabolite to another. Two different injections with a different acquisition mode maximized the fragmentation of metabolites (Section 3.4.2.). Data-dependent MS/MS acquisition with dynamic exclusion and apex triggering enabled us to obtain a few MS/MS spectra for each chromatographic peak and allowed molecules with a less intense signal intensity to also trigger MS/MS spectra.

## 3. Material and Methods

### 3.1. Chemicals and Reagents

α-PHP (1-phenyl-2-(1′-pyrrolidinyl)-1-hexanone) and 4F-α-PVP (1-(4′′-fluorophenyl)-2-(1′-pyrrolidinyl)-1-pentanone) standards were purchased from Cayman Chemical (Ann Arbor, MI, USA), and diclofenac standard was acquired from Toronto Research Chemicals (Toronto, ON, Canada). All the standards were dissolved in methanol to 1 mg/mL and stored at −20 °C until analysis. Water, formic acid, and methanol (Optima™ LC/MS) were from Fisher Scientific (Fair Lawn, NJ, USA), and acetonitrile was obtained from Sigma-Aldrich^®^ (St. Louis, MO, USA). All the solvents and reagents were LC-MS grade. Ten-donor-pooled cryopreserved human hepatocytes and Krebs–Henseleit buffer (KHB) were purchased from BioreclamationIVT (Baltimore, MD, USA).

### 3.2. Incubation with Pooled Human Hepatocytes

Incubations with human hepatocytes were conducted following our on-site workflow, as previously described [45,46]. Briefly, the hepatocytes were thawed at 37 °C and solubilized in KHB to 2 × 10^6^ viable cells/mL. Two hundred fifty microliters cell suspension was gently mixed with 250 µL of 20 µmol/L α-PHP or 4F-α-PVP in KHB and incubated at 37 °C for 0 or 3 h. Reactions were quenched with 500 µL acetonitrile and incubates were stored at −80 °C until analysis. Diclofenac was incubated under the same conditions to ensure proper metabolic activity. Negative controls with α-PHP or 4F-α-PVP in KHB without hepatocytes, and with KHB and hepatocytes without the drugs were incubated simultaneously. Each incubation was performed once due to the high cost of pooled human hepatocytes, but positive and negative controls were included to document appropriate metabolic activity and control for potential interference and contribution from the blank. The advantages and limitations of this approach are further discussed in two recent review articles [47,48].

### 3.3. Sample Preparation

Incubates were thawed at room temperature and vortex mixed. One hundred microliters was vortex mixed with 100 µL acetonitrile and centrifuged at 4 °C, 15,000× *g*, for 10 min. Supernatants were evaporated to dryness at 40 °C, and the residues were reconstituted in 150 µL LC mobile phases A:B 80:20 (*v/v*). After centrifugation at 4 °C, 15,000× *g*, for 5 min, supernatants were transferred into LC autosampler vials with glass inserts.

### 3.4. LC-HRMS/MS Parameters

LC-HRMS/MS analysis was performed on a Dionex Ultimate™ 3000 chromatography system coupled with a Thermo Scientific Q Exactive™ (Fremont, CA, USA) Plus mass spectrometer with a heated electrospray ionization (ESI) interface operating in positive-ion mode. During method optimization, α-PHP and 4F-α-PVP did not produce any signal when ESI was operated in negative-ion mode, and their metabolites were likely to produce a signal in positive-ion mode. Data reprocessing was performed in Ancona, Italy, on a Dionex Ultimate™ 3000 coupled with a Q Exactive™ (Ancona, Italy) combined with heated ESI operating in the same conditions.

#### 3.4.1. LC Parameters

Samples were kept at 15 °C in an autosampler and 15 µL was injected onto the chromatographic system. Separation was achieved on a Synergi™ Hydro-RP column (100 × 2.0 mm, 4.0 µm; Phenomenex, Torrance, CA, USA) equipped with a guard cartridge of identical stationary phase (10 × 2.1 mm; Phenomenex) that was maintained at 30 °C throughout the analysis. A 30 min elution was performed with 0.1% formic acid in water (mobile phase A) and 0.1% formic acid in acetonitrile (mobile phase B) at a 0.4 mL/min flow rate—the gradient started with 2% B held for 2 min, was increased to 95% B within 18 min, and was held at 95% B for 5 min, before returning to initial conditions within 1 min, followed by a 4-min equilibration. LC efflux was diverted to waste the first 2 min and the last 5 min of the gradient.

#### 3.4.2. HRMS/MS Parameters

Source parameters were optimized by post-column infusion of α-PHP and 4F-α-PVP standards in LC mobile phases A:B 50:50 (*v/v*) at 0.5 mL/min. Source parameters for α-PHP incubates were: spray voltage, 4 kV; sheath gas flow rate, 50 arbitrary units (a.u.); auxiliary gas flow rate, 5 a.u.; sweep gas flow rate, 2 a.u.; S-lens radio frequency level, 50 a.u.; auxiliary gas heater temperature, 400 °C; capillary temperature, 300 °C. Source parameters for 4F-α-PVP incubates were: spray voltage, 4 kV; sheath gas flow rate, 40 a.u.; auxiliary gas flow rate, 5 a.u.; sweep gas flow rate, 2 a.u.; S-lens radio frequency level, 50 a.u.; auxiliary gas heater temperature, 500 °C; capillary temperature, 300 °C. Injections were performed in duplicate with two different acquisition modes to obtain orthogonal spectral information.

In a first injection, data were acquired in full-scan MS (FullMS) and data-dependent MS/MS (ddMS^2^) modes, which triggered MS/MS data acquisitions based on the intensity of the ions detected in FullMS scans and an inclusion list of expected metabolites. FullMS settings were: resolution, 70,000; scan time, 2–25 min; mass range, *m*/*z* 100–500; automatic gain control (AGC) target, 1 × 10^6^; max injection time (IT), 200 ms. ddMS^2^ settings were: resolution, 17,500; topN, 5 (pick others if idle); intensity threshold, 2 × 10^4^; isolation window, *m*/*z* 1.5; normalized collision energy (NCE), 40%, 50%, and 60% (35%, 50%, and 65% for 4F-α-PVP); apex triggering, 3–6 s; dynamic exclusion, 2 s; AGC target, 1 × 10^5^; max IT, 50 ms. Inclusion lists were generated based on prior metabolite identification studies [21,22,23,49] and postulation (Table 2).

In a second injection, data were acquired in FullMS/all-ion fragmentation (AIF)/ddMS^2^ mode, which triggered MS/MS data acquisitions based on specific neutral losses detected in FullMS and AIF scans. FullMS and ddMS^2^ settings were the same as described above. Resolution, intensity threshold, mass range, NCE, AGC target, and max IT were the same as those of ddMS^2^. Neutral loss lists were generated based on the fragmentation pattern of parent drugs, i.e., α-PHP or 4F-α-PVP, in the conditions of the analysis, and including glucuronide and sulfate losses in case of phase II metabolism (Table 2).

### 3.5. Metabolite Identification

Raw data files from 0 and 3 h incubations and controls were processed with Compound Discoverer™ (Thermo Scientific; Ancona, Italy) and compiled in a single analysis. Retention times of the chromatographic peaks were aligned following an adaptive curve model with a 5-ppm mass tolerance and a 0.1-min maximum time shift. Mass tolerance for MS identification was 5 ppm, with a 13,000 intensity threshold (20,000 for 4F-α-PVP) and a 50% intensity tolerance for isotopes. Mass tolerance for fragment identification was 10 ppm, with a signal-to-noise ratio higher than 3. Chromatographic peaks detected in controls with a similar or higher intensity than α-PHP and 4F-α-PVP incubations were filtered out. Detected peaks were compared to a list of theoretical molecules automatically generated by a combination of probable metabolic transformations, following the settings displayed in Table 3.

## 4. Conclusions

We have provided the first metabolic profile of 4F-α-PVP and identified an additional major metabolite of α-PHP using human hepatocyte incubations, LC-HRMS/MS analysis, and data-mining software. We suggest α-PHP dihydroxy-pyrrolidinyl (P2), α-PHP hexanol (P3), α-PHP 2′-keto-pyrrolidinyl-hexanol (P4), and α-PHP d2′-keto-pyrrolidinyl (P5) as markers of α-PHP use, and 4F-α-PVP dihydroxy-pyrrolidinyl (F2), 4F-α-PVP hexanol (F3), 4F-α-PVP 2′-keto-pyrrolidinyl-hexanol (F4), and 4F-α-PVP d2′-keto-pyrrolidinyl (F5) as markers of 4F-α-PVP use. The identification of this new α-PHP metabolite (P2) may be critical to prove α-PHP use in clinical or forensic settings and to further substantiate findings from hepatic microsome investigations. These results must be confirmed with further experiments using suitable reference standards. Additionally, these are also valuable data for standard manufacturers to focus their synthesis efforts. Further pharmacokinetic and pharmacodynamic studies should be performed to better understand the effects of α-PHP and 4F-α-PVP.

## Figures and Tables

**Table 1:** Accurate mass molecular ion, retention time (RT), elemental composition, nominal mass for diagnostic product ions, MS peak areas, and α-PHP and 4F-α-PVP metabolites peak area fraction (sum = 95 and 98%, respectively) in hepatocyte incubations. Peak area for α-PHP and 4F-α-PVP at T_0h_ were 6.1 × 10^7^ and 7.5 × 10^7^, respectively. Metabolites are listed by ascending RT. MS, mass spectrometry; ND, not detected.

ID	Biotransformation	[M + H]^+^ (*m*/*z*)	RT (min)	Mass Error (ppm)	Elemental Composition	Diagnostic Product Ions (*m*/*z*)	Peak Area at T_3h_	Metabolites Peak Area Fraction (%)
α-PHP	Parent drug	246.1848	9.38	1.79	C_16_H_23_NO	70, 91, 105, 119, 140, 175	1.3 × 10^7^	
P1	Hydroxylation (α-PHP 2′-hydroxypyrrolidinyl)	262.1798	8.70	1.32	C_16_H_23_NO_2_	70, 91, 105, 119, 156, 175	6.1 × 10^5^	2.6%
P2	Dihydroxylation (α-PHP dihydroxy-pyrrolidinyl)	278.1746	9.02	1.72	C_16_H_23_NO_3_	87, 91, 105, 118, 174, 186	1.0 × 10^7^	44%
P3	β-Ketoreduction (α-PHP hexanol)	248.2005	9.66	1.63	C_16_H_25_NO	72, 91, 104, 117, 173, 230	9.7 × 10^6^	40%
P4	β-Ketoreduction + Ketone formation (α-PHP 2′-ketopyrrolidinyl-hexanol)	262.1799	12.52	0.97	C_16_H_23_NO_2_	70, 86, 91, 98, 117, 159	1.3 × 10^6^	5.3%
P5	Ketone formation (α-PHP 2′-ketopyrrolidinyl)	260.1642	14.77	1.00	C_16_H_21_NO_2_	86, 91, 105, 129, 133, 175	7.0 × 10^5^	2.9%
4F-α-PVP	Parent drug	250.1598	8.71	1.33	C_15_H_20_FNO	70, 84, 109, 123, 126, 179	2.2 × 10^7^	
F1	Hydroxylation (4F-α-PVP 2′-hydroxypyrrolidinyl)	266.1547	8.12	1.35	C_15_H_20_FNO_2_	70, 109, 123, 142, 179, 248	6.4 × 10^5^	2.8%
F2	Dihydroxylation (4F-α-PVP dihydroxy-pyrrolidinyl)	282.1496	8.46	1.42	C_15_H_20_FNO_3_	87, 109, 123, 136, 178, 179	9.4 × 10^6^	41%
F3	β-Ketoreduction (4F-α-PVP hexanol)	252.1755	8.94	1.18	C_15_H_22_FNO	72, 109, 122, 190, 191, 234	9.1 × 10^6^	40%
F4	β-Ketoreduction + Ketone formation (4F-α-PVP 2′-ketopyrrolidinyl-hexanol)	266.1548	11.70	1.01	C_15_H_20_FNO_2_	86, 98, 109, 135, 163, 248	1.7 × 10^6^	7.4%
F5	Ketone formation (4F-α-PVP 2′-ketopyrrolidinyl)	264.1391	14.00	1.15	C_15_H_18_FNO_2_	98, 109, 123, 140, 161, 179	1.6 × 10^6^	7.1%

**Table 2:** Inclusion and neutral loss lists for the FullMS/ddMS2 and FullMS/AIF/ddMS2 acquisitions, respectively, used during HRMS analysis of hepatocytes incubations. FullMS/ddMS2, full-scan mass spectrometry/data-dependent tandem mass spectrometry; FullMS/AIF/ddMS2, full-scan mass spectrometry/all-ion fragmentation/data-dependent tandem mass spectrometry; HRMS, high resolution mass spectrometry.

**α-PHP.**					
**FullMS/ddMS^2^ acquisition**					
**[M + H]^+^ (*m*/*z*)**	**Formula**	**[M + H]^+^ (*m*/*z*)**	**Formula**	**[M + H]^+^ (*m*/*z*)**	**Formula**
192.1383	C_12_H_17_NO	260.1645	C_16_H_21_NO_2_	280.1907	C_16_H_25_NO_3_
208.1332	C_12_H_17_NO_2_	262.1802	C_16_H_23_NO_2_	292.1543	C_16_H_21_NO_4_
244.1696	C_16_H_21_NO	264.1958	C_16_H_25_NO_2_	296.1856	C_16_H_25_NO_4_
**246.1852**	**C_16_H_23_NO**	276.1594	C_16_H_21_NO_3_	342.1370	C_16_H_23_NSO_5_
248.2009	C_16_H_25_NO	278.1751	C_16_H_23_NO_3_	438.2122	C_22_H_31_NO_8_
**FullMS/AIF/ddMS^2^ acquisition**					
**Neutral loss (*m*/*z*)**	**Fragment loss**	**Neutral loss (*m*/*z*)**	**Fragment loss**	**Neutral loss (*m*/*z*)**	**Fragment loss**
−57.0704	−C_4_H_9_	−127.1361	−C_8_H_17_N	−174.1044	−C_12_H_14_O
−71.0735	−C_4_H_9_N	−141.1517	−C_9_H_19_N	−176.1201	−C_12_H_16_O
−79.9563	−SO_3_	−155.1310	−C_9_H_17_NO	−176.0315	−C_6_H_8_O_6_
−106.0418	−C_7_H_6_O	−162.1044	−C_11_H_14_O		
**4F-α-PVP**					
**FullMS/ddMS^2^ acquisition**					
**[M + H]^+^ (*m*/*z*)**	**Formula**	**[M + H]^+^ (*m*/*z*)**	**Formula**	**[M + H]^+^ (*m*/*z*)**	**Formula**
194.1176	C_11_H_15_NO_2_	252.1758	C_15_H_22_FNO	282.1700	C_15_H_23_NO_4_
196.1132	C_11_H_14_FNO	262.1438	C_15_H_19_NO_3_	284.1657	C_15_H_22_FNO_3_
212.1081	C_11_H_14_FNO_2_	264.1394	C_15_H_18_FNO_2_	296.1293	C_15_H_18_FNO_4_
232.1696	C_15_H_21_NO	264.1594	C_15_H_21_NO_3_	300.1606	C_15_H_22_FNO_4_
246.1489	C_15_H_19_NO_2_	266.1551	C_15_H_20_FNO_2_	328.1213	C_15_H_21_NSO_5_
248.1445	C_15_H_18_FNO	268.1707	C_15_H_22_FNO_2_	346.1119	C_15_H_20_FNSO_5_
248.1645	C_15_H_21_NO_2_	278.1387	C_15_H_19_NO_4_	424.1966	C_21_H_29_NO_8_
**250.1602**	C_15_H_20_FNO	280.1344	**C_15_H_18_FNO_3_**	442.1872	C_21_H_28_FNO_8_
250.1802	C_15_H_23_NO_2_	282.1500	C_15_H_20_FNO_3_		
**FullMS/AIF/ddMS^2^ acquisition**					
**Neutral loss (*m*/*z*)**	**Fragment loss**	**Neutral loss (*m*/*z*)**	**Fragment loss**	**Neutral loss (*m*/*z*)**	**Fragment loss**
−43.0548	−C_3_H_7_	−124.0325	−C_7_H_5_FO	−176.0315	−C_6_H_8_O_6_
−71.0735	−C_4_H_9_N	−127.1361	−C_8_H_17_N	−178.0794	−C_11_H_11_FO
−79.9563	−SO_3_	−141.1154	−C_8_H_15_NO	−180.0951	−C_11_H_13_FO
−113.1205	−C_7_H_15_N	−166.0794	−C_10_H_11_FO		

Bold indicates parent drugs.

**Table 3:** Compound Discoverer™ processing settings for identifying α-PHP and 4F-α-PVP metabolites.

	α-PHP	4F-α-PVP
Phase I expected transformations	Dehydrogenation (–2H), dihydrodiol formation (+2O +2H), oxidation (+O), oxidative deamination to alcohol (–N +O –H), oxidative deamination to ketone (–N +O –3H), reduction (+2H)	Dehydrogenation (–2H), dihydrodiol formation (+2O +2H), oxidation (+O), oxidative deamination to alcohol (–N +O –H), oxidative deamination to ketone (–N +O –3H), oxidative defluorination (–F +O +H), reduction (+2H), reductive defluorination (–F +H)
Phase II expected transformations	Acetylation (+2C +O +2H), glucuronidation (+6C +6O +10H), sulfation (+S +3O)	Acetylation (+2C +O +2H), glucuronidation (+6C +6O +10H), sulfation (+S +3O)
Maximum number of dealkylation steps	2	2
Maximum number of phase II reactions	1	1
Maximum number of reactions	5	5

## Data Availability

Data is contained within the article.

## Abbreviations

SC	Synthetic cathinone
NPS	Novel psychoactive substance
DAT	Dopamine transporter
NET	Norepinephrine transporter
SERT	Serotonin transporter
MDMA	3″,4″-Methylenedioxymethamphetamine
MDPV	Methylenedioxypyrovalerone
α-PHP	α-pyrrolidinohexaphenone
4F-α-PVP	4″-Fluoro-α-pyrrolidinovalerophenone
α-PVP	α-Pyrrolidinovalerophenone
MDPH	3″,4″-Methylenedioxy-α-pyrrolidinohexiophenone
EMCDDA	European Monitoring Centre for Drugs and Drug Addiction
UNODC	United Nations Office on Drugs and Crime
WHO	World Health Organization
PV8	α-Pyrrolidinoheptaphenone
α-PVT	α-Pyrrolidinopentiothiophenone
LC	Liquid chromatography
MS	Mass spectrometry
MS/MS	Tandem mass spectrometry
HRMS	High resolution mass spectrometry
GC	Gas chromatography
FullMS	Full scan
ddMS^2^	Data-dependent tandem mass spectrometry
AIF	All-ion fragmentation
AGC	Automatic gain control
IT	Injection time
